# Analysis of substitution rates showed that TLR5 is evolving at different rates among mammalian groups

**DOI:** 10.1186/s12862-019-1547-4

**Published:** 2019-12-02

**Authors:** Ana Pinheiro, Ana Águeda-Pinto, José Melo-Ferreira, Fabiana Neves, Joana Abrantes, Pedro J. Esteves

**Affiliations:** 1 CIBIO-UP, Centro de Investigação em Biodiversidade e Recursos Genéticos, Universidade do Porto, InBIO, Laboratório Associado, Campus Agrário de Vairão, 4485-661 Vairão, Portugal; 2 Departamento de Biologia, Faculdade de Ciências, Universidade do Porto, 4169-007 Porto, Portugal; 3 Center for Immunotherapy, Vaccines, and Virotherapy (CIVV), The Biodesign Institute, Arizona State University, Tempe, AZ 85287 USA; 4 CITS - Centro de Investigação em Tecnologias de Saúde, CESPU, 4585-116 Gandra, Portugal

**Keywords:** TLRs, Substitution rate, Relaxed selection, Evolution

## Abstract

**Background:**

Toll-like receptors (TLRs) are the most widely studied innate immunity receptors responsible for recognition of invading pathogens. Among the TLR family, TLR5 is the only that senses and recognizes flagellin, the major protein of bacterial flagella. TLR5 has been reported to be under overall purifying selection in mammals, with a small proportion of codons under positive selection. However, the variation of substitution rates among major mammalian groups has been neglected. Here, we studied the evolution of TLR5 in mammals, comparing the substitution rates among groups.

**Results:**

In this study we analysed the TLR5 substitution rates in Euungulata, Carnivora, Chiroptera, Primata, Rodentia and Lagomorpha, groups. For that, Tajima’s relative rate test, Bayesian inference of evolutionary rates and genetic distances were estimated with CODEML’s branch model and RELAX. The combined results showed that in the Lagomorpha, Rodentia, Carnivora and Chiroptera lineages TLR5 is evolving at a higher substitution rate. The RELAX analysis further suggested a significant relaxation of selective pressures for the Lagomorpha (K = 0.22, *p* < 0.01), Rodentia (K = 0.58, *p* < 0.01) and Chiroptera (K = 0.65, *p* < 0.01) lineages and for the Carnivora ancestral branches (K = 0.13, *p* < 0.01).

**Conclusions:**

Our results show that the TLR5 substitution rate is not uniform among mammals. In fact, among the different mammal groups studied, the Lagomorpha, Rodentia, Carnivora and Chiroptera are evolving faster. This evolutionary pattern could be explained by 1) the acquisition of new functions of TLR5 in the groups with higher substitution rate, i.e. TLR5 neofunctionalization, 2) by the beginning of a TLR5 pseudogenization in these groups due to some redundancy between the TLRs genes, or 3) an arms race between TLR5 and species-specific parasites.

## Background

One of the first steps of an immune response involves the recognition of invading pathogens. The innate immunity receptors responsible for this recognition are collectively called pattern recognition receptors (PRRs). PRRs recognize structural moieties of the pathogens antigens shared by infectious agents but distinguishable from host molecules, called pathogen associated molecular patterns (PAMPs) and also molecules released by damaged cells, called Damage-Associated Molecular Patterns (DAMPs) [1, 2]. PAMP recognition allows PRRs to distinguish between self and non-self. In mammals, PRRs are divided into four major types: 1) nucleotide-binding oligomerization domain (NOD)-like receptors and 2) retinoid acid inducible genes (RIG)-like receptors, which are found on the cytoplasm of host cells, and 3) C-type lectin receptors (CTLRs) and 4) Toll-like receptors (TLRs), which are bound to cell membranes. Of these, the TLRs are the most widely studied.

TLRs are type I transmembrane glycoproteins which can be expressed either in the cell surface or intracellular compartments. To date, 13 mammalian TLRs have been identified and together these recognize a wide repertoire of pathogens, including bacteria, fungi, protozoa and viruses [3]. TLRs may locate on the cell surface or in the endosome. TLR1, TLR2, TLR5, TLR6 and TLR10 are located on the cell surface and recognize bacterial, fungal and parasite ligands. TLR3, TLR7, TLR8, TLR9, TLR11, TLR12 and TLR13 are located in the endosomal membrane and recognize mostly viral nucleic acids but also bacterial molecules and *Toxoplasma gondii* profiling like molecule. TLR4 locates both on the cell surface and endosome and recognizes bacterial and viral ligands (reviewed in [4]). Mammalian immune system genes are permanently engaged in a co-evolutionary arms race with their target pathogens and hence are expected to have fast evolutionary rates. Despite TLRs being evolutionary conserved proteins across vertebrates, patterns of positive selection have been described on these genes regions responsible for pathogen detection [5–8].

TLR5 is the only TLR that senses and recognizes flagellin, the major protein of bacterial flagella [9]. After flagelin recognition, TLR5 triggers the immunologic responses for the clearance of the pathogen [9, 10]. More recently, the TLR5 has also been shown to regulate the composition of intestinal microbiota and to protect the liver against chronic inflammation through flagellin recognition (reviewed in [11]). In mammals, TLR5 has been reported to be under overall purifying selection, with a small proportion of codons under positive selection [6, 8, 12].

Despite the large number of existing studies on TLRs evolution and function, the variation of substitution rates between major mammalian groups has been disregarded. The differences in substitution rates among major mammalian groups are particularly interesting since it was recently shown that the Lagomorpha TLR2 has a substitution rate higher than all the other mammalian groups [13]. Here, we studied the evolution of TLR5 in mammals comparing the substitution rates estimated for different groups of mammals. The results showed that the Primata and the Euungulata are evolving slower than the other mammalian groups.

## Results

**Fig. 1:**
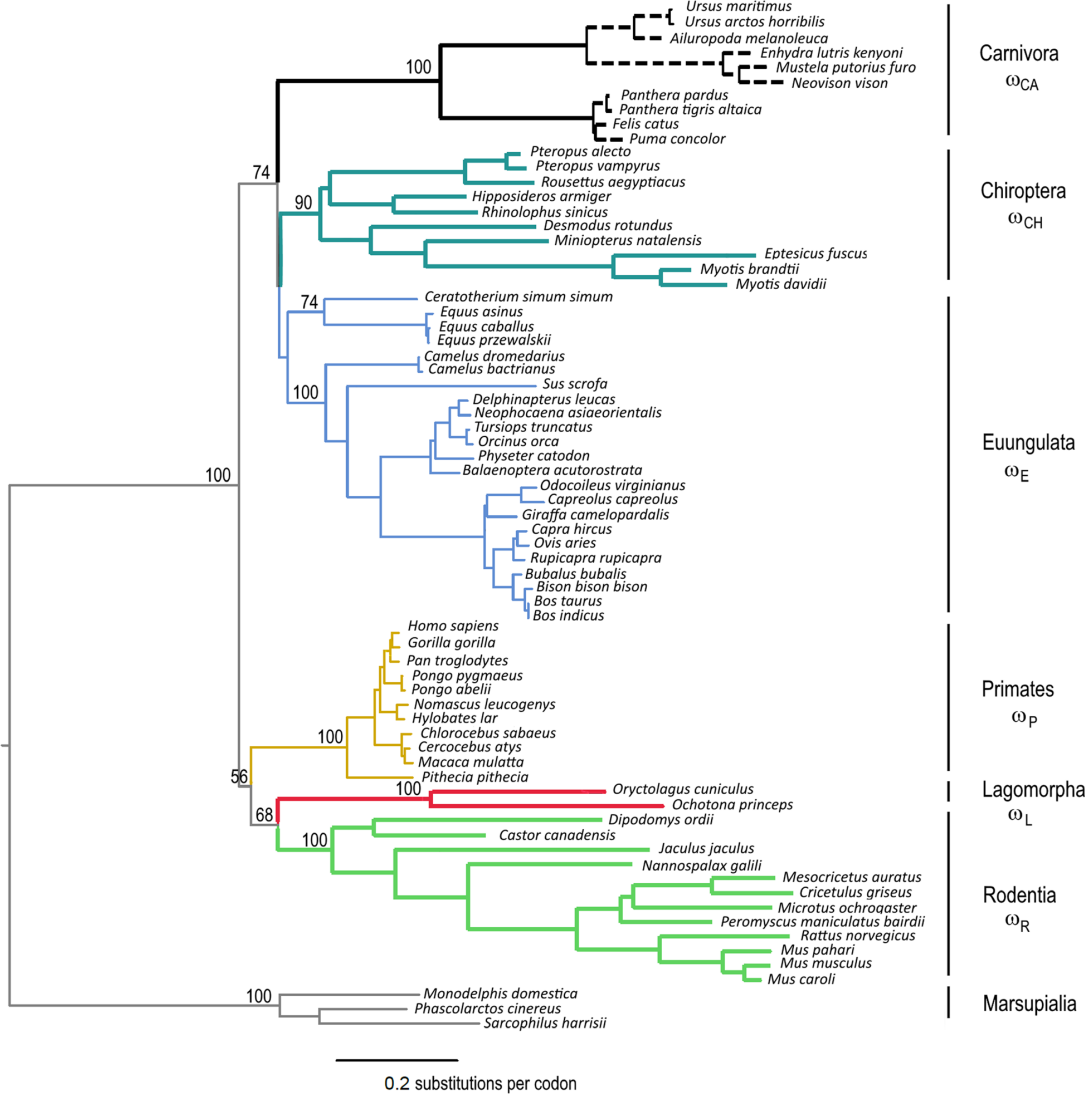
Phylogenetic tree of the TLR5 gene. Maximum likelihood (ML) method and the GTR + G model of nucleotide substitution were used to obtain the TLR5 phylogenetic tree, which was then constrained to better recover the mammalian relationships according to the currently accepted mammalian species tree [14]. Branch lengths in substitutions per codon were calculated under the CODEML M0 model [15]. Highlighted in bold and colored are the foreground branches used in branch model and RELAX analyses: Chiroptera in dark green, Lagomorpha in red, Rodentia in green and Carnivora in black; in dashed bold are the Carnivora tips, excluded from the Carnivora ancestral analysis in RELAX. Colored are also the Euungulate (light blue) and Primata (yellow) branches

The observation that in the TLR5 phylogeny (Fig. 1) some mammalian groups, such as the Lagomorpha, Rodentia, Chiroptera and Carnivora, present longer branches when compared to Primata and Euungulata suggested that in the different mammalian groups, *TLR5* is evolving at different paces. To test our hypothesis, we first performed the Tajima’s Relative Rate test, which counts the number of changes that occurred in two species relatively to an outgroup. The null hypothesis of equal rates among lineages is then compared to the alternative hypothesis of different rates among lineages through a likelihood ratio test. We tested the differences between representative species of the studied eutherian mammalian order’s and either human (*Homo sapiens*) or cow (*Bos taurus*), using as outgroup the marsupial koala (*Phascolarctos cinereus*). For species representative of the Lagomorpha, Rodentia, Chiroptera and Carnivora orders the statistically significant test *p*-value (*p* < 0,05; Table 1) allowed rejecting the null hypothesis of equal rates among lineages.

**Table 1:** *P*-values obtained in Tajima’s Relative Rate Test using human or cow as reference and the marsupial koala as outgroup

Taxon B	Taxon A – Human (*H. sapiens*)	Taxon A- Cow (*B. Taurus*)
*Oryctolagus cuniculus*	< 0.01	< 0.01
*Mus musculus*	< 0.01	< 0.01
*B. taurus*	0.10	–
*Orcinus orca*	0.19	0.30
*Sus scrofa*	< 0.01	0.19
*Equus caballus*	0.21	0.38
*E. fuscus*	< 0.01	< 0.01
*Pteropus alecto*	< 0.01	< 0.05
*Mustela putorius*	< 0.01	< 0.01
*Panthera tigris*	< 0.01	< 0.01

**Fig. 2:**
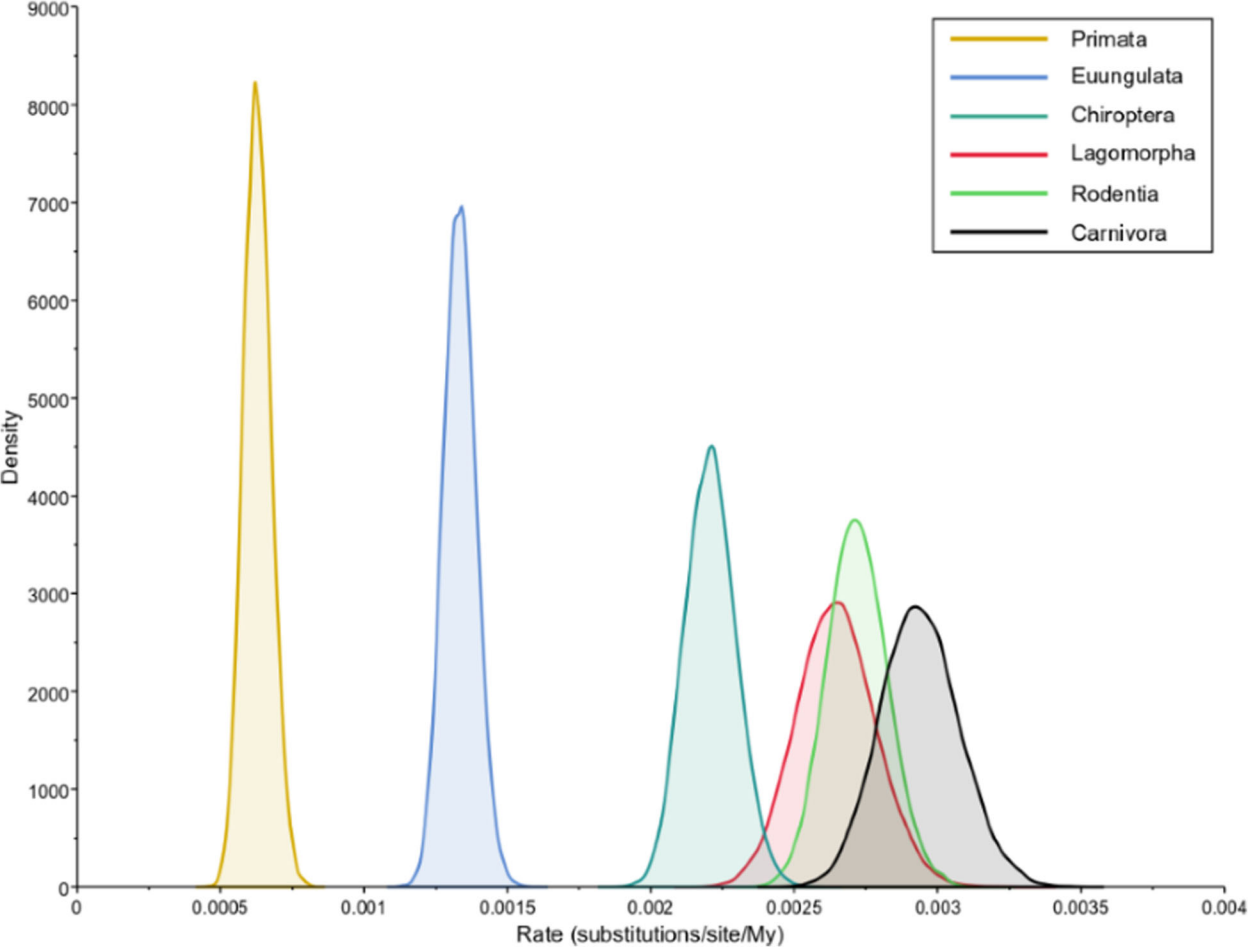
Bayesian estimates of evolutionary rates for mammalian lineages. Posterior density distribution of the inferred evolutionary rates for the six studied mammalian lineages: Chiroptera, Lagomorpha, Rodentia, Carnivora, Euungulate and Primata. The rates were inferred using BEAST software [16] under a fixed local clock model [17]

Furthermore, a Bayesian inference of evolutionary rates for each of these mammalian lineages also showed that the Carnivora (0.0027–0.0032 substitutions/site/million years, 95% Highest Posterior Density (HPD) interval), Rodentia (0.0025–0.0029, 95% HPD interval), Lagomorpha (0.0024–0.0029, 95% HPD interval) and Chiroptera (0.0020–0.0024, 95% HPD interval) have substantially higher substitution rates than Primata (0.0005–0.0007, 95% HPD interval) or Euungulata (0.0012–0.0014, 95% HPD interval) (Fig. 2).

The calculated genetic distances are also higher for the Lagomorpha, Rodentia, Chiroptera and Carnivora groups (highest genetic distances of 0.176, 0.253, 0.223 and 0.202, respectively; Table 2) compared to the Primata or Euungulata lineages (highest genetic distances of 0.067 and 0.155, respectively; Table 2). Considering that the divergence times, i.e. time since the last common ancestor, are older for Primata and Euungulata (71.5 million years ago (mya) and 80 mya, respectively [18]) than for Lagomorpha, Rodentia, Chiroptera and Carnivora (50.2 mya, 69 mya, 66.5 mya and 54.7 mya, respectively [18]), the calculated genetic distances also suggest that these latter lineages are evolving at a higher rate than Primata and Euungulata.

**Table 2:** TLR5 range of genetic distances calculated for mammalian groups

Mammalian lineage	nucleotide distance	amino acid distance
Primata	0.002–0.067	0.002–0.056
Euungulata	0.002–0.155	0.000–0.219
Rodentia	0.022–0.253	0.023–0.299
Lagomorpha	0.176	0.209
Carnivora	0.003–0.202	0.003–0.236
Chiroptera	0.017–0.223	0.023–0.251

Considering the obtained indications that for some mammalian branches the *TLR5* sequence is evolving at a higher rate we proceeded to investigate the selection rates of *TLR5* in mammals. For that, we first tested several branch models in CODEML. The null model of a single ω ratio for all branches was rejected against the alternative models of two, five and seven ω ratios (Tables 3 and 4). The model with five ω ratios along the phylogenetic tree, i.e., one ω ratio for each of the Lagomorpha, Rodentia, Chiroptera and Carnivora branches and a single ω ratio for the remaining branches, was significantly better than the two ω ratios model and was not rejected against the more complex seven ω ratios model (Tables 3 and 4), suggesting that the selective pressure has changed for each one of the lineages showing long branches on the phylogenetic tree.

**Table 3:** lnL values and parameters estimates under CODEML different branch models

Model	ω_0_	ω_L_	ω_R_	ω_CH_	ω_CA_	ω_P_	ω_E_	p	lnL
*M0*	0.3375	= ω_0_	= ω_0_	= ω_0_	= ω_0_	= ω_0_	= ω_0_	144	−46,458.51
*M2*	0.4084	0.3063	= ω_L_	= ω_L_	= ω_L_	= ω_0_	= ω_0_	145	−46,439.89
*M5*	0.4087	0.2503	0.3321	0.3184	0.2677	= ω_0_	= ω_0_	148	−46,432.80
*M7*	0.3759	0.2505	0.3322	0.3184	0.2678	0.4322	0.4057	150	−46,431.47

p, number of parameters in the model; ω_L_, ω_R_, ω_CH_, ω_CA_, ω_P_, ω_E_, ω_0_ are the d_N_/d_S_ ratios for branches Lagomorpha, Rodentia, Chiroptera, Carnivora, Primata, Euungulata and all other branches, respectively (see Fig. 1)

**Table 4:** Likelihood ratio statistics for testing hypotheses

Null hypothesis	Alternative hypothesis	df	2ΔlnL
*M0*	*M2*	1	37.25***
*M0*	*M5*	4	51.42***
*M0*	*M7*	6	54.08***
*M2*	*M5*	3	14.17**
*M2*	*M7*	5	16.83**
*M5*	M7	3	2.66

*df* degrees of freedom; **, *p* < 0.01; ***, *p* < 0.001

**Fig. 3:**
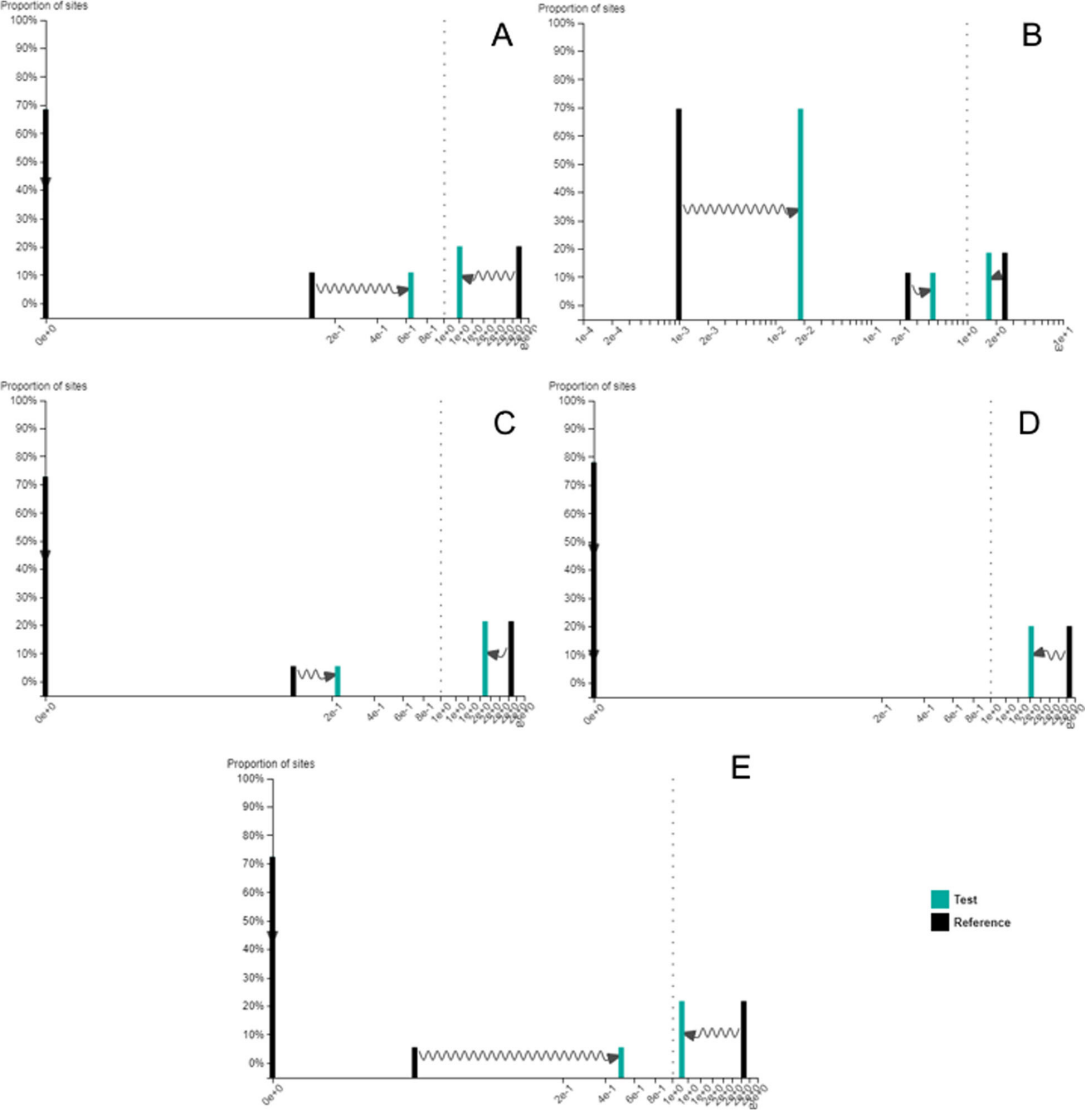
ω distributions under the RELAX alternative model. Test group is shown in green and reference branches (Primata, Euungulata, Marsupialia) are shown in black. **a** Lagomorpha as test branch **b**) Rodentia as test branch **c**) Chiroptera as test branch, **d**) Carnivora as test branch and **e**) Carnivora ancestral branches as test branch

The RELAX analysis further suggested a relaxation of selective pressures for these long branched lineages with estimated K of 0.22 (*p* < 0.01) for the Lagomorpha, 0.58 (p < 0.01) for the Rodentia and 0.65 (p < 0.01) for the Chiroptera, with a reduction in the strength of both purifying and positive selection acting on these lineages TLR5 (Fig. 3, Additional file 1: Tables S1, S2 and S3). For the Carnivora lineage a reduction in the relative strength of positive selection was identified (Fig. 3) which, however, was not significant (K = 0.54, *p* = 1.00) (Additional file 1: Table S4). Defining as test branches only the Carnivora ancestral branches (see Fig. 1 for the ancestral branches selected as test branches) the RELAX analysis detected a significant relaxation of selective pressure (K = 0.13, *p* < 0.01) with a reduction in the strength of both purifying and positive selection for the Carnivora ancestral branches (Fig. 3, Additional file 1: Table S5).

The higher substitution rate associated with a relaxation of selective pressure inferred for the Lagomorpha, Rodentia, Chiroptera and Carnivora ancestrals TLR5 could translate into changes in the *TLR5* structure in these groups. However, the analysis of the *TLR5* structure is overall conserved among the different mammalian groups, with an ectodomain comprising 17–21 Leucine rich repeats (LRRs) and one LRR-CT, or two in Euungulata, a transmembrane and TIR domains (data not shown), with no significant alterations in the structure of the long branched lineages.

## Discussion

The incidence of positive selection on *TLR5* evolution has been studied in some mammalian groups [6–8, 12] but a curious aspect of the mammalian TLR5 phylogeny has been neglected, which motivated this study. Indeed, in the TLR5 phylogeny (Fig. 1) some mammalian groups, such as the Lagomorpha, Rodentia, Chiroptera and Carnivora, present longer branches when compared to Primata and Euungulata. The observed differences in the branch lengths suggested that in the different mammalian groups, TLR5 is evolving at different paces.

To test our hypothesis, we performed a batch of tests on the alignment and phylogenetic tree of the mammalian TLR5 sequences. The results obtained all show that there are, in fact, different evolutionary rates for the TLR5 of different mammalian lineages and that in the Lagomorpha, Rodentia, Chiroptera and Carnivora groups the TLR5 sequence is evolving at a higher rate compared to Primata and Euungulata. The Tajima’s Relative Rate test indicated that the Lagomorpha, Rodentia, Chiroptera and Carnivora lineages have a different substitution rate than Primata and Euungulata and the Bayesian inference of evolutionary rates and the calculated genetic distances show that the Lagomorpha, Rodentia, Chiroptera and Carnivora lineages have higher substitution rates compared to Primata and Euungulata. Genetic distances are useful to estimate divergence times between species and populations, with typically the highest genetic distance representing the highest evolutionary divergence [19]. Considering the older divergence times of Primata and Euungulata (71.5 million years ago (mya) and 80 mya, respectively [18]) it was expected to obtain higher genetic distances for TLR5 sequences in these lineages. Instead, these lineages genetic distances were lower than those obtained for the Lagomorpha, Rodentia, Chiroptera and Carnivora lineages (time since the first common ancestor 50.2 mya, 69 mya, 66.5 mya and 54.7 mya, respectively [18]), thus showing evidence that the substitution rate is higher for the long branched lineages.

TLR5 has been reported to be under overall purifying selection in mammals, with a small proportion of codons under positive selection in Artiodactyls [6], Primata [8] and Carnivores [12]. Since TLR5 is the only member of the TLR family to recognize flagellin it would be reasonable to expect it to be evolving under functional constraint, and hence the strong signal of purifying selection, but the positively selected codons may reflect some species specific adaptation to pathogens [20]. The evidence of relaxation of positive selective strength we have obtained for Lagomorpha, Rodentia, Chiroptera and Carnivora ancestrals TLR5, indicates that these signatures of species specific adaptation to pathogens are being lost in these clades. A relaxation of selective strength may occur in several contexts among which the removal of a functional constraint, an environmental change that may remove or weaken a source of selection, or after gene duplication as selection may be relaxed on one of the copies leading to pseudogenization or neofunctionalization (reviewed in [21]). Since there is no evidence of pseudogenization of the Lagomorpha, Rodentia, Chiroptera and Carnivora *TLR5*, we suggest that in these clades TLR5 may have an alternative function. In fact, TLR5 function has been suggested to be altered in the dog [22], and in humans the segregation at high frequency (up to 23%) of a TLR5 stop substitution suggests that it is functionally redundant [8]. In birds, several TLR5 lineages have undergone pseudogenization events [23]. More recently, TLR5 was found to mediate touch sensation being expressed by peripheral sensory neurons (reviewed in [11]).

The TLR family has evolved by gene duplication, gene loss and gene conversion events, that have originated the TLR functional diversification and different TLR repertoire in vertebrate species [24]. To date, 13 TLRs have been identified in mice of which only 10 are present in humans (reviewed in [4]) and in rabbit the TLR7 is absent [25, 26]. The variation in the TLR repertoire in vertebrate species illustrates that different gene combinations constitute efficient systems to respond to pathogens, where genes of recognized relevant function are lost others fulfill the same role. The presence of other proteins that are able to interact with flagellin might also explain the observed relaxation of selective pressure in Lagomorpha, Rodentia and Chiroptera TLR5. A recent study showed that in mice TLR11 can also recognize flagellin [27]. Despite the interaction between flagellin and TLR11 is more restricted than its interaction with TLR5, flagellin can interact with TLR11 using both N- and C- domains [27]. Moreover, flagellin, as well as the bacterial protein T3SS rod, are also recognized by another PRR, the NLRC4, which is differently activated by pathogenic or commensal bacteria and plays an important role in host defense [28, 29]. The finding that different proteins can interact with flagellin might suggest that the action of TLR5 is not essential to induce an immune response in the host, ultimately resulting in a relaxation of the selective pressure in TLR5.

## Conclusions

Our results clearly show that the TLR5 substitution rate is not uniform among mammals. The Lagomorpha, Rodentia, Carnivora and Chiroptera are evolving faster than the other main mammal groups. This evolutionary pattern could be explained by 1) the acquisition of new functions of TLR5 in the groups with higher substitution rate, i.e. TLR5 neofunctionalization, 2) by the beginning of a TLR5 pseudogenization in these groups due to some redundancy between the TLRs genes, or 3) an arms race between TLR5 and species specific parasites.

## Methods

### Sequences

Publicly available sequences for mammalian *TLR5* were obtained from GenBank (http://www.ncbi.nlm.nih.gov/genbank/). In total, 71 species representative of Artiodactyla, Cetacea, Perissodactyla, Carnivora, Chiroptera, Primata, Rodentia, Lagomorpha, and Marsupialia, were included in the analyses (accession numbers are given in Additional file 1: Table S6). Sequences were aligned using CLUSTAL W [30] as implemented in BioEdit [31], and corrected manually. The obtained alignment is given in Additional file 2.

### Evolutionary analysis

MEGA version X software [32] was used to reconstruct the mammalian *TLR5* phylogenetic tree using the maximum likelihood (ML) method and the GTR + G model of nucleotide substitution. Node support was determined from 1000 bootstrap replicate ML trees. Tajima’s relative rate tests [33] were performed to assess the statistical significance of the different evolutionary rates of *TLR5* in mammalian groups using MEGA version X software [32]. This software was also used to calculate the nucleotide distances using the maximum composite likelihood method, uniform rates among sites, heterogeneous rates among lineages and pairwise deletion of gaps options and the amino acid distances using the p-distance method, uniform rates among sites, heterogeneous rates among lineages and pairwise deletion of gaps options.

The evolutionary rates were further inferred using the Bayesian method implemented in BEAST v1.10.4 [16] under a fixed local clock model [17]. This relaxed clock allows variation of evolutionary rates among monophyletic lineages. The analysis was calibrated setting normally distributed priors for the time of the most recent common ancestor of seven monophyletic clades, with mean estimated by Tarver et al. [34] – Boreotheria, 85.07 million years ago (Mya); Euungulata, 71.35 Mya; Primata, 69.27 Mya; Rodents, 61.97 Mya; Chiroptera, 58.23 Mya; Carnivora, 52.61 Mya; and Lagomorphs, 48.57 Mya – and a standard deviation of 2. Posterior probabilities were determined using the Yule tree prior and a GTR + G nucleotide substitution model. Independent runs of 10,000,000 generations were performed, and convergence was assessed using Tracer v1.7 [35]. Final estimates were based on the combined results of three replicate runs, discarding the first 10% as burn-in.

We next investigated the selection rates of *TLR5* in mammals using the branch model analysis of CODEML program of the PAML 4.7 package [15], and performed the RELAX analysis [36] available in the Data Monkey web server (http://www.datamonkey.org/relax). For these analyses the obtained *TLR5* gene tree was constrained (Fig. 1) to better recover the mammalian relationships according to the currently accepted mammalian species tree [14]. To identify lineages with accelerated evolution we tested diverse branch models on CODEML, considering one to seven ω ratios. Based on the hypothesis that mammalian lineages showing long branches on the phylogenetic tree are evolving at distinct evolutionary pressures from the remaining mammalian lineages, our two rate model compared these long branched lineages to the rest. The five rate model allowed for a different ω ratio for each of the lagomorphs, rodents, bats and carnivores lineages against a single ω ratio for the background and the seven rate model allowed each lineage to have a ω ratio. A summary of the tested models is given in Table 5; the labeled trees are given in Additional file 3. Models were compared using a likelihood ratio test (2ΔlnL) and test significance was obtained by using a χ2 distribution under the corresponding degrees of freedom. Each branch model was run with three different initial ω ratio values to ensure models convergence to stable maximum likelihoods. To further assess the strength of natural selection acting on each of the long branch lineages we next performed the RELAX analysis. RELAX is a tool that can determine whether a test branch/branches is under relaxed or intensified selective strength relative to the reference branch/branches [36]. This is achieved by comparing the null model, in which k (selection intensity parameter) is constrained to 1, against the alternative model, in which k is a free parameter, through a likelihood ratio test. A significant k < 1 means there has been a relaxation of selection for the test branches whereas k > 1 means the test branches are under intensified selection [36]. The RELAX analyses were performed targeting each one of the four long branched lineages, i.e. Lagomorpha, Rodentia, Chiroptera and Carnivora, using the Primata, Euungulata (Artiodactyla, Cetacea and Perissodactyla) and Marsupialia branches as reference.

**Table 5:** Different tested branch models

Model	ω ratios
*M0*	one ratio: ω_0_ = ω_L_ = ω_R_ = ω_CH_ = ω_CA_ = ω_P_ = ω_E_
*M2*	two ratio: ω_0_ = ω_P_ = ω_E_, ω_L_ = ω_R_ = ω_CH_ = ω_CA_
*M5*	five ratio: ω_0_ = ω_P_ = ω_E_, ω_L_, ω_R_, ω_CH_, ω_CA_
*M7*	seven ratio: ω_0_, ω_P_, ω_E_, ω_L_, ω_R_, ω_CH_, ω_CA_

ω_0,_ background ω ratio; ω_P_, Primata ω ratio; ω_E_, Euungulata ω ratio; ω_L_, Lagomorpha ω ratio; ω_R_, Rodentia ω ratio; ω_CH_, Chiroptera ω ratio; ω_CA,_ Carnivora ω ratio

The different mammalian species TLR5 structure was determined using the program LRRfinder [37] to identify the different TLR domains. LRR finder is a webserver that identifies conserved regions of leucine rich repeats, LRRs, in given sequences as well as other TLR domains such as the signal peptide, LRR N- and C- terminus, the transmembrane and the TIR domains.

## Supplementary information

**Additional file 1:** **Tables S1-S5.** Tests for Selection Relaxation. **Table S6.** Genbank accession numbers of TLR5 sequences used in this study.

**Additional file 2.** Alignment of TLR5 sequences for the mammalian species used in this study

**Additional file 3.** Labeled trees used in the CODEML branch tests.

## Data Availability

All data analysed during this study is included in this published article [and its supplementary information files].

## Abbreviations

CTL: C-type lectin
DAMPs: Damage-associated molecular patterns
LRR-CT: c-terminal LRR
LRRs: Leucine rich repeats
ML: Maximum likelihood
mya: million years ago
NLRC4: NOD-Like Receptor C4
NOD: Nucleotide-binding oligomerization domain
PAMPs: Pathogen associated molecular patterns
PRRs: Pattern recognition receptors
RIG: Retinoid acid inducible genes
TIR: Toll/interleukin 1 resistance
TLRs: Toll-like receptors
