# Supplementary material for: Analysis of substitution rates showed that TLR5 is evolving at different rates among mammalian groups
Source: BMC Evol Biol. 2019 Dec 2;19:221. doi: 10.1186/s12862-019-1547-4 (PMC6889247; doi:10.1186/s12862-019-1547-4)
Supplement: Supplementary file 1 — Additional file 1: Tables S1-S5. Tests for Selection Relaxation. Table S6. Genbank accession numbers of TLR5 sequences used in this study. [file 12862_2019_1547_MOESM1_ESM.docx]

**Table S1– Test for Selection Relaxation on the Chiroptera branch**. Log likelihood values and parameter estimates for the RELAX analysis.  *log L*, log likelihood value under the model; *p*, number of parameters in the model; *AICc*, small-sample correction Akaike information criterion; Branch set, indicates which branch set each parameter belongs to; *ω*1, first omega rate class; *ω*2, second omega rate class; *ω*3, third omega rate class. Test for selection relaxation (*K* = 0.32) was significant (*P* < 0.001, LR = 13.78)

| Model | *log L* | *P* | *AICc* | Branch Set | ω1 | ω2 | ω3 |
| --- | --- | --- | --- | --- | --- | --- | --- |
| General descriptive | -45174.8 | 297 | 90946.4 | Shared | 0.00 (69.42%) | 0.99 (29.68%) | 6.80 (0.90%) |
| Relax alternative | -45309.4 | 164 | 90947.8 | Test | 0.00 (72.90%) | -0.22 (5.61%) | 1.70 (21.49%) |
|  |  |  |  | Reference | 0.00 (72.90%) | 0.10 (5.61%) | 2.26 (21.49%) |
| Relax null | -45316.3 | 163 | -90959.5 | Test | 0.01 (74.16%) | 0.25 (5.85%) | 2.12 (19.99%) |
|  |  |  |  | Reference | 0.01 (74.16%) | 0.25 (5.85%) | 2.12 (19.99%) |
| Relax partitioned descriptive | -45308.5 | 168 | 90954.0 | Test | 0.00 (74.04%) | 0.37 (5.09%) | 1.72 (20.87%) |
|  |  |  |  | Reference | 0.07 (72.14%) | 0.35 (15.50%) | 3.18 (12.36%) |

**Table S2 – Test for Selection Relaxation on the Lagomorpha branch.** Log likelihood values and parameter estimates for the RELAX analysis.  *log L*, log likelihood value under the model; *p*, number of parameters in the model; *AICc*, small-sample correction Akaike information criterion; Branch set, indicates which branch set each parameter belongs to; *ω*1, first omega rate class; *ω*2, second omega rate class; *ω*3, third omega rate class. Test for selection relaxation (*K* = 0.22) was significant (*P* < 0.01, LR = 8.15).

| Model | *log L* | *P* | *AICc* | Branch Set | ω1 | ω2 | ω3 |
| --- | --- | --- | --- | --- | --- | --- | --- |
| General descriptive | -45180.3 | 297 | 90957.4 | Shared | 0.00 (68.20%) | 0.99 (31.09%) | 12.39 (0.71%) |
| Relax alternative | -45313.8 | 164 | 90956.6 | Test | 0.00 (68.59%) | 0.64 (11.10%) | 1.21 (20.32%) |
|  |  |  |  | Reference | 0.00 (68.59%) | 0.13 (11.10%) | 2.35 (20.32%) |
| Relax null | -45317.9 | 163 | 90962.7 | Test | 0.09 (65.85%) | 0.47 (27.99%) | 4.65 (6.16%) |
|  |  |  |  | Reference | 0.09 (65.85%) | 0.47 (27.99%) | 4.65 (6.16%) |
| Relax partitioned descriptive | -45311.9 | 168 | 90960.8 | Test | 0.13 (64.85%) | 0.24 (29.11%) | 2.93 (6.04%) |
|  |  |  |  | Reference | 0.03 (66.72%) | 0.73 (26.98%) | 4.58 (6.30%) |

**Table S3 – Test for Selection Relaxation on the Rodentia branch.** Log likelihood values and parameter estimates for the RELAX analysis. *log L*, log likelihood value under the model; *p*, number of parameters in the model; *AICc*, small-sample correction Akaike information criterion; Branch set, indicates which branch set each parameter belongs to; *ω*1, first omega rate class; *ω*2, second omega rate class; *ω*3, third omega rate class. Test for selection relaxation (*K* = 0.33) was significant (*P* < 0.01, LR = 7.87)

| Model | *log L* | *P* | *AICc* | Branch Set | ω1 | ω2 | ω3 |
| --- | --- | --- | --- | --- | --- | --- | --- |
| General descriptive | -45166.6 | 297 | 90930.1 | Shared | 0.00 (72.60%) | 1.00 (25.69%) | 5.73 (1.71%) |
| Relax alternative | -45313.6 | 164 | 90956.1 | Test | 0.02 (69.67%) | 0.44 (11.60%) | 1.69 (18.73%) |
|  |  |  |  | Reference | 0.00 (69.67%) | 0.24 (11.60%) | 2.48 (18.73%) |
| Relax null | -45317.6 | 163 | 90962.0 | Test | 0.08 (66.21%) | 0.54 (27.99%) | 4.28 (5.81%) |
|  |  |  |  | Reference | 0.08 (66.21%) | 0.54 (27.99%) | 4.28 (5.81%) |
| Relax partitioned descriptive | -45310.0 | 168 | 90956.9 | Test | 0.01 (60.72%) | 0.69 (36.87%) | 5.61 (2.40%) |
|  |  |  |  | Reference | 0.01 (66.27%) | 0.79 (27.80%) | 4.64 (5.93%) |

**Table S4 – Test for Selection Relaxation on the Carnivora branch.** Log likelihood values and parameter estimates for the RELAX analysis. *log L*, log likelihood value under the model; *p*, number of parameters in the model; *AICc*, small-sample correction Akaike information criterion; Branch set, indicates which branch set each parameter belongs to; *ω*1, first omega rate class; *ω*2, second omega rate class; *ω*3, third omega rate class. Test for selection relaxation (*K* = 0.54) was not significant (*P* > 0.1, LR = -20.32)

| Model | *log L* | *P* | *AICc* | Branch Set | ω1 | ω2 | ω3 |
| --- | --- | --- | --- | --- | --- | --- | --- |
| General descriptive | -45166.8 | 297 | 90930.4 | Shared | 0.00 (72.68%) | 1.00 (25.63%) | 6.21 (1.69%) |
| Relax alternative | -45349.7 | 164 | 91028.2 | Test | 0.00 (1.76%) | 0.00 (78.09%) | 1.62 (20.16%) |
|  |  |  |  | Reference | 0.00 (1.76%) | 0.00 (78.09%) | 2.46 (20.16%) |
| Relax null | -45339.5 | 163 | 91005.9 | Test | 0.00 (64.79%) | 1.00 (34.86%) | 55.10 (0.35%) |
|  |  |  |  | Reference | 0.00 (64.79%) | 1.00 (34.86%) | 55.10 (0.35%) |
| Relax partitioned descriptive | -45297.5 | 168 | 90932.0 | Test | 0.00 (72.78%) | 1.00 (26.91%) | 780.29 (0.31%) |
|  |  |  |  | Reference | 0.00 (64.51%) | 1.00 (33.41%) | 8.59 (2.08%) |

**Table S5 – Test for Selection Relaxation on the Carnivora ancestral branches.** Log likelihood values and parameter estimates for the RELAX analysis. *log L*, log likelihood value under the model; *p*, number of parameters in the model; *AICc*, small-sample correction Akaike information criterion; Branch set, indicates which branch set each parameter belongs to; *ω*1, first omega rate class; *ω*2, second omega rate class; *ω*3, third omega rate class. Test for selection relaxation (*K* = 0.26) was significant (*P* < 0.01, LR = 28.31).

| Model | *log L* | *P* | *AICc* | Branch Set | ω1 | ω2 | ω3 |
| --- | --- | --- | --- | --- | --- | --- | --- |
| General descriptive | -45171.9 | 297 | 90940.7 | Shared | 0.00 (70.41%) | 0.99 (28.47%) | 3.90 (1.12%) |
| Relax alternative | -45307.6 | 164 | 90944.1 | Test | 0.00 (75.16%) | 0.89 (6.44%) | 1.26 (18.39%) |
|  |  |  |  | Reference | 0.00 (75.16%) | 0.65 (6.44%) | 2.46 (18.39%) |
| Relax null | -45321.8 | 163 | 90970.4 | Test | 0.00 (76.43%) | 0.85 (7.09%) | 2.31 (16.48%) |
|  |  |  |  | Reference | 0.00 (76.43%) | 0.85 (7.09%) | 2.31 (16.48%) |
| Relax partitioned descriptive | -45306.9 | 168 | 90950.8 | Test | 0.00 (75.70%) | 1.00 (0.00%) | 1.20 (24.30%) |
|  |  |  |  | Reference | 0.06 (74.67%) | 0.45 (12.53%) | 3.09 (12.79%) |

**Table S6 - Genbank accession numbers of TLR5 sequences used in this study.**

| **Species** | **Common name** | **Order-Family** | **Accession number** |
| --- | --- | --- | --- |
| *Homo sapiens* | Human | Primates - Hominidae | DQ026419.1 |
| *Pan troglodytes* | Chimpanzee | Primates - Hominidae | KF320788.1 |
| *Gorilla gorilla* | Western lowland gorilla | Primates - Hominidae | KF320781.1 |
| *Pongo pygmaeus* | Bornean orangutan | Primates - Hominidae | AB445649.1 |
| *Pongo abelii* | Sumatran orangutan | Primates - Hominidae | XM_024255862.1 |
| *Nomascus leucogenys* | Northern white-cheeked gibbon | Primates - Hylobatidae | XM_012506470.1 |
| *Hylobates lar* | Lar gibbon | Primates - Hylobatidae | EU488851.1 |
| *Macaca mulatta* | Rhesus macaque | Primates - Cercopithecidae | NM_001130429.1 |
| *Cercocebus atys* | Sooty mangabey | Primates - Cercopithecidae | NM_001305947.1 |
| *Chlorocebus sabaeus* | Green monkey | Primate - Cercopithecidae | XM_007988358.1 |
| *Pithecia pithecia* | White-faced Saki | Primates - Pitheciidae | FJ542214.1 |
| *Oryctolagus cuniculus* | European rabbit | Lagomorpha - Leporidae | NM_001329083.1 |
| *Ochotona princeps* | American pika | Lagomorpha -Ochotonidae | XM_004578616.2 |
| *Castor canadensis* | American beaver | Rodentia - Castoridae | XM_020173768.1 |
| *Dipodomys ordii* | Ord's Kangaroo Rat | Rodentia - Heteromyidae | XM_013014975.1 |
| *Jaculus jaculus* | lesser Egyptian jerboa | Rodentia - Dipodidae | XM_004653238.2 |
| *Nannospalax galili* | Northern Israeli blind subterranean mole rat | Rodentia - Spalacidae | XM_008823748.2 |
| *Mesocricetus auratus* | Syrian hamster | Rodentia - Cricetidae | XM_021231939.1 |
| *Cricetulus griseus* | Chinese hamster | Rodentia - Cricetidae | XM_003494963.1 |
| *Peromyscus maniculatus bairdii* | North American deermouse | Rodentia - Cricetidae | XM_016007067.1 |
| *Microtus ochrogaster* | Prairie vole | Rodentia - Cricetidae | XM_013346964.1 |
| *Mus pahari* | Gairdner's shrewmouse | Rodentia - Muridae | XM_021199413.1 |
| *Mus musculus* | House mouse | Rodentia - Muridae | XM_017321698.1 |
| *Rattus norvegicus* | Brown rat | Rodentia - Muridae | FJ750588.1 |
| *Mus caroli* | Ryukyu Mouse | Rodentia - Muridae | XM_021175380.1 |
| *Rupicapra rupicapra* | Chamois | Artiodactyla - Bovidae | JQ811845.1 |
| *Capra hircus* | Goat | Artiodactyla - Bovidae | KF731670.1 |
| *Bubalus bubalis* | Water buffalo | Artiodactyla - Bovidae | GU903502.1 |
| *Bos taurus* | European cattle | Artiodactyla - Bovidae | JQ805125.1 |
| *Bos indicus* | Zebu | Artiodactyla - Bovidae | GQ866979.1 |
| *Bison bison bison* | Plains bison | Artiodactyla - Bovidae | XM_010852829.1 |
| *Ovis aries* | Domestic sheep | Artiodactyla - Bovidae | NM_001135926.1 |
| *Giraffa camelopardalis* | Giraffe | Artiodactyla - Giraffidae | JQ811844.1 |
| *Odocoileus virginianus* | White-tailed deer | Artiodactyla - Cervidae | XM_020879522.1 |
| *Capreolus capreolus* | European Roe Deer | Artiodactyla - Cervidae | KM488255.1 |
| *Sus scrofa* | wild boar | Artiodactyla - Suidae | FJ754217.1 |
| *Camelus dromedarius* | Camel | Artiodactyla - Camelidae | XM_010992762.1 |
| *Camelus bactrianus* | Bactrian camel | Artiodactyla - Camelidae | XM_010973980.1 |
| *Balaenoptera acutorostrata* | Minke whale | Artiodactyla - Cetacea - Balaenopteridae | XM_007172037.1 |
| *Physeter catodon* | Sperm whale | Artiodactyla - Cetacea - Physeteridae | XM_007121463.2 |
| *Delphinapterus leucas* | White whale | Artiodactyla - Cetacea - Monodontidae | XM_022579286.1 |
| *Tursiops truncatus* | Common bottlenose dolphin | Artiodactyla - Cetacea - Delphinidae | XM_019920593.1 |
| *Orcinus orca* | Killer whale | Artiodactyla - Cetacea - Delphinidae | [XM_012533577.1](https://www.ncbi.nlm.nih.gov/nucleotide/XM_012533577.1?report=genbank&log$=nuclalign&blast_rank=9&RID=TY73551T015) |
| *Neophocaena asiaeorientalis* | Narrow-ridged finless porpoise | Artiodactyla - Cetacea - Phocoenidae | XM_024750465.1 |
| *Equus caballus* | Horse | Perissodactyla-Equidae | XM_023632733.1 |
| *Equus przewalskii* | Przewalski's horse | Perissodactyla-Equidae | XM_008533391.1 |
| *Equus asinus* | Donkey | Perissodactyla-Equidae | XM_014838730.1 |
| *Ceratotherium simum simum* | White rhinoceros | Perissodactyla - Rhinocerotidae | XM_004439536.2 |
| *Eptesicus fuscus* | Big brown bat | Chiroptera - Vespertilionidae | XM_028158022.1 |
| *Myotis brandtii* | Brandt's bat | Chiroptera - Vespertilionidae | XM_014539936.1 |
| *Myotis davidii* | David’s bat | Chiroptera - Vespertilionidae | XM_015562369.1 |
| *Miniopterus natalensis* | Natal long-fingered bat | Chiroptera - Miniopteridae | XM_016221152.1 |
| *Desmodus rotundus* | Common vampire bat | Chiroptera - Phyllostomidae | XM_024575982.1 |
| *Hipposideros armiger* | Great roundleaf bat | Chiroptera - Hipposideridae | XM_019639666.1 |
| *Rhinolophus sinicus* | Chinese Horseshoe Bat | Chiroptera - Rhinolophidae | XR_002139551.1 |
| *Pteropus alecto* | Black fruit bat | Chiroptera - Pteropodidae | NM_001320280.1 |
| *Pteropus vampyrus* | Large flying fox | Chiroptera - Pteropodidae | XM_011360853.1 |
| *Rousettus aegyptiacus* | Egyptian fruit bat | Chiroptera - Pteropodidae | XM_016122698.1 |
| *Panthera pardus* | Leopard | Carnivora - Felidae | XM_019436125.1 |
| *Panthera tigris altaica* | Tiger | Carnivora - Felidae | XM_015544459.1 |
| *Felis catus* | Cat | Carnivora - Felidae | XM_023247432.1 |
| *Puma concolor* | Cougar | Carnivora - Felidae | XM_025926314.1 |
| *Enhydra lutris kenyoni* | Sea otter | Carnivora - Mustelidae | XM_022508525.1 |
| *Mustela putorius furo* | Ferret | Carnivora - Mustelidae | XM_013050635.1 |
| *Neovison vison* | American mink | Carnivora - Mustelidae | KU133954.1 |
| *Ursus maritimus* | Polar Bear | Carnivora - Ursidae | XM_008695476.1 |
| *Ursus arctos horribilis* | Grizzly bear | Carnivora - Ursidae | XM_026509475.1 |
| *Ailuropoda melanoleuca* | Giant panda | Carnivora - Ursidae | XM_019802948.1 |
| *Monodelphis domestica* | Gray short-tailed opossum | Didelphimorphia - Didelphidae | XM_001376152.2 |
| *Phascolarctos cinereus* | Koala | Diprotodontia - Phascolarctidae | KP792550.1 |
| *Sarcophilus harrisii* | Tasmanian devil | Marsupialia - Dasyuridae | XM_003767727.3 |
